# A Procedure for Designing Custom-Made Implants for Forehead Augmentation in People Suffering from Apert Syndrome

**DOI:** 10.1007/s10916-020-01611-9

**Published:** 2020-07-27

**Authors:** Marco Mandolini, Manila Caragiuli, Agnese Brunzini, Alida Mazzoli, Mario Pagnoni

**Affiliations:** 1 Department of Industrial Engineering and Mathematical Sciences, Università Politecnica delle Marche, Via Brecce Bianche 12, 60131 Ancona, Italy; 2 Department of Materials, Environmental Sciences and Urban Planning, Università Politecnica delle Marche, Via Brecce Bianche 12, 60131 Ancona, Italy; 3 Mario Pagnoni, yourFACE, Clinica Parioli, Via Felice Giordano 8, 00197 Rome, Italy

**Keywords:** Rapid prototyping, Craniomaxillofacial surgery, Computer-aided design, Medical devices, Implant design

## Abstract

This paper presents a methodological procedure, based on the anatomical reconstruction and constrained deformation, to design custom-made implants for forehead augmentation in people affected by Apert syndrome, experiencing a frontal bone deficiency. According to the anthropometric theory, a cranial landmarks identification procedure was applied to retrieve, from a repository, a healthy skull, used as reference geometry for implant modelling. Then, using constrained deformation and free-form modelling techniques, it was possible to design a patient-specific implant. At last, the implant was realised using a custom mould, specially designed according to the patient’s needs to provide an accurate fit of the defect site. The design procedure was tested on a patient suffering from Apert syndrome. Three implants were virtually modelled and 3D-printed for pre-surgical evaluation. Their shapes were 3D compared with a reference one (handcrafted by a surgeon) to test the accuracy. Deviations are negligible, and the customised implant fulfilled the surgeon’s requirements.

## Introduction

Craniofacial skeleton reconstruction is challenging, primarily when congenital diseases affect the symmetry of the skull with complex and irregular defects. Computer-Aided Design (CAD) and Computer-Aided Manufacturing (CAM) technologies have replaced traditional techniques based on manual shaping [1] and casting [2], providing tools for prostheses customisation. Thus, patient-specific implants enable to get an accurate fit of the anatomical site by aligning the tapered edges to the adjacent bone boundaries [3], providing better post-operative outcomes and craniofacial aesthetic improvements.

Depending on the lesion complexity (size and location), the aesthetic recovery, the functional requirements and the technical considerations established by the surgeon, different design techniques are available for the reconstruction of craniomaxillofacial defects:

- *Mirrored imaging technique*: this method consists of mirroring the sound side of the skull over the contralateral part and subsequently apply a Boolean difference to get the implant. This method is suitable for skulls with low asymmetry and unilateral lesions [4–6] and for significant defects which do not cross the midline.
- *Thin plate spline (TPS) interpolation or deformation*: this method comprises interpolation functions able to generate an approximation of the skull surface at the lesion site by warping and deforming a target based on two sets of homologous points defined over both a reference model and a target. Carr et al. [7] showed their potential in a cranial defect reconstruction. This approach can be suitable for defects spanning across the midface by using an average template skull [8]. Being surface interpolators, TPS is not convenient when dealing with extensive defect areas.
- *Anatomical reconstruction or free form modelling*: the implant can be designed by using a supporting geometry, for instance, the residual geometry of the patient’s bone, and free-form modelling tools such as lines, planes and curves provided by CAD software.
- *Template-based technique*: an average skull or a similar skull is used as a reference to design the implant geometry by establishing a spatial correspondence between the lesioned area on the patient skull and its corresponding fragment on a reference model. Singare et al. [9] designed a frontal prosthesis by choosing a reference skull from a CT database. The approach is suitable for highly asymmetric skulls and large and complex defects, even spanning the midline.
- *Snakes method*: 2D CT images of the skull are pre-processed to extract the inner and outer bone contour. Mathematical curves are used to fit the bone contours, and then by stacking all the slices, it is possible to reconstruct the skull defect. The reconstruction accuracy can be improved by using a 3D multi-grid snake, also resulting in a reduction of the processing time from 3 h to 20 min [10]. This approach requires care in the presence of frontal bone damages because of the higher curvature of the region and does not reconstruct significant defects accurately.

All the previously listed approaches have never been used before for designing custom made implants for people suffering from Apert syndrome. This is a genetic condition characterised by craniosynostosis (the premature fusion of the skull sutures), midface hypoplasia (the underdevelopment of the midfacial region), exophthalmos (protruding eyeballs because of a decreased orbital volume), syndactyly of the hands and feet at the cutaneous and bony level and varying degrees of neurocognitive impairment. In particular, the premature fusion of the coronal suture interferes with the skull healthy growing resulting in a peaked head and a prominent flat forehead, a condition described as acrobrachycephaly [11].

In the present paper, authors propose a new design methodology to reconstruct a cranial bone deficiency of patients affected by Apert syndrome (i.e., forehead augmentation). Such methodology was defined considering eight criteria, listed in Table 1 (i.e., “*user intervention*”, “*defect size*”, “*lesion site*”, “*operational time*”, “*degree of skull asymmetry*”, “*skills*”, “*set-up required*” and “*quality control*”). According to the characteristics of the Apert pathology, where the patient’s skull may by highly deformed, “*defect size*” and “*degree of skull asymmetry*” were the most relevant criteria considered when defining the proposed design methodology. Since people suffering from Apert need custom-made implants for restoring their aesthetics, the implant precision should be as high as possible. For this reason, “*quality control*” criteria (i.e., “*curvature*”, “*tangency*” and “*continuity*”) have been considered of high importance. Criteria related to operational procedures (“*set-up required*”, “*user intervention*”, “*operational time*” and “*skills*”) have been considered less critical since this paper is focused on the definition of a design procedure. Its implementation in a 3D CAD tool is beyond the aim of the present work.

**Table 1:** Evaluation metrics

Evaluation metrics	3 Points	6 Points	9 Points
Defect site	Only unilateral	Uni- or bilateral, but not beyond midface	Uni- or bilateral and/or beyond the midface
**Defect size** (we assume that a technique able to reconstruct a large defect is suitable also for moderate and small defects)	Small (<25 cm^2^)	Moderate (25–200 cm^2^)	Large (>200 cm^2^)
**Degree of skull asymmetry** (the more significant the asymmetry, the higher the deformation, the more the difficulty in the reconstruction, the more powerful is the method)	Low	Medium	High
**Set-up required** (preliminary activities required to implement the method, e.g., creation of a set of skull CT scans, the definition of the symmetry plane, identification of anatomical landmarks)	Yes	–	No (the CT scans of the skull were not accounted as set-up required)
**User intervention** (automation degree of a procedure: the more automated the approach, the lower the user intervention, the higher the score)	High	Medium	Low
**Operational time** (time required to accomplish the design of the implant)	High	Medium	Low
**Skills** (expertise required to carry out the implant design)	High	Medium	Low
**Quality control**			
Curvature	Poor	Adequate	Optimal
Tangency	No	–	Yes
Continuity	No	–	Yes

Table 2 summarises scores and weights assigned by the authors to each design methodology and criterion. It is worth noting that the *mirroring approaches* cannot be applied to reconstruct the typical Apert defects due to the lesion site and the degree of cranial asymmetry. *TPS* and *Snake* methods hardly provide the proper shape of the implant when the defect is in a region of high curvature. *Free-form modelling* cannot offer a standard procedure to tackle the design of such a patient-tailored implant since it relies on ad hoc modelling depending on the case under study, resulting not applicable to other cranial defects. The 3D modelling procedure is not supported by any reference geometry, and the result is strongly subjective since it depends on the skill of the technician who is modelling the implant. *Template-based* methods do not rely on the patient’s anatomy and do not ensure tangency at the implant-bone interface since they are prone to alignment errors.

**Table 2:** Comparative evaluation of cranial defects reconstruction techniques (weighted scores within rounded squares)

Evaluation metrics	*Weight*	Mirroring	Thin-plate spline deformation (TPS)	Free-form modelling	Template-based	Snake
Defect site	*0.00*	3 (0.00)	9 (0.00)	9 (0.00)	9 (0.00)	9 (0.00)
Defect size	*0.20*	6 (1.20)	3 (0.60)	6 (1.20)	9 (1.80)	6 (1.20)
Degree of skull asymmetry	*0.20*	3 (0.60)	9 (1.80)	6 (1.20)	9 (1.80)	6 (1.20)
Set-up required	*0.10*	3 (0.30)	3 (0.30)	9 (0.90)	3 (0.30)	6 (0.60)
User intervention	*0.05*	6 (0.30)	3 (0.15)	3 (0.15)	3 (0.15)	9 (0.45)
Operational time	*0.05*	6 (0.30)	3 (0.15)	3 (0.15)	6 (0.30)	9 (0.45)
Skills	*0.10*	6 (0.60)	6 (0.60)	3 (0.30)	6 (0.60)	6 (0.60)
Quality						
Curvature Tangency Continuity	*0.10* *0.10* *0.10*	9 (0.90) 3 (0.30) 3 (0.30)	3 (0.30) 9 (0.90) 9 (0.90)	6 (0.60) 9 (0.90) 9 (0.90)	9 (0.90) 3 (0.30) 3 (0.30)	3 (0.30) 3 (0.30) 9 (0.90
Total score	***1.00***	**48 (4.80)**	**57 (5.70)**	**63 (6.30)**	**60 (6.45)**	**66 (6.00)**

The method proposed in the present paper wants to combine benefits of *template-based* methods (adoption of reference geometries, required for managing high skull asymmetry and large defect size) and *free-form modelling* methods (high control of the implant quality). Thus, a methodological procedure based on template deformation and anatomical reconstruction was devised and tested on a patient affected by Apert syndrome experiencing a frontal bone deficiency.

## Methods

**Fig. 1:**
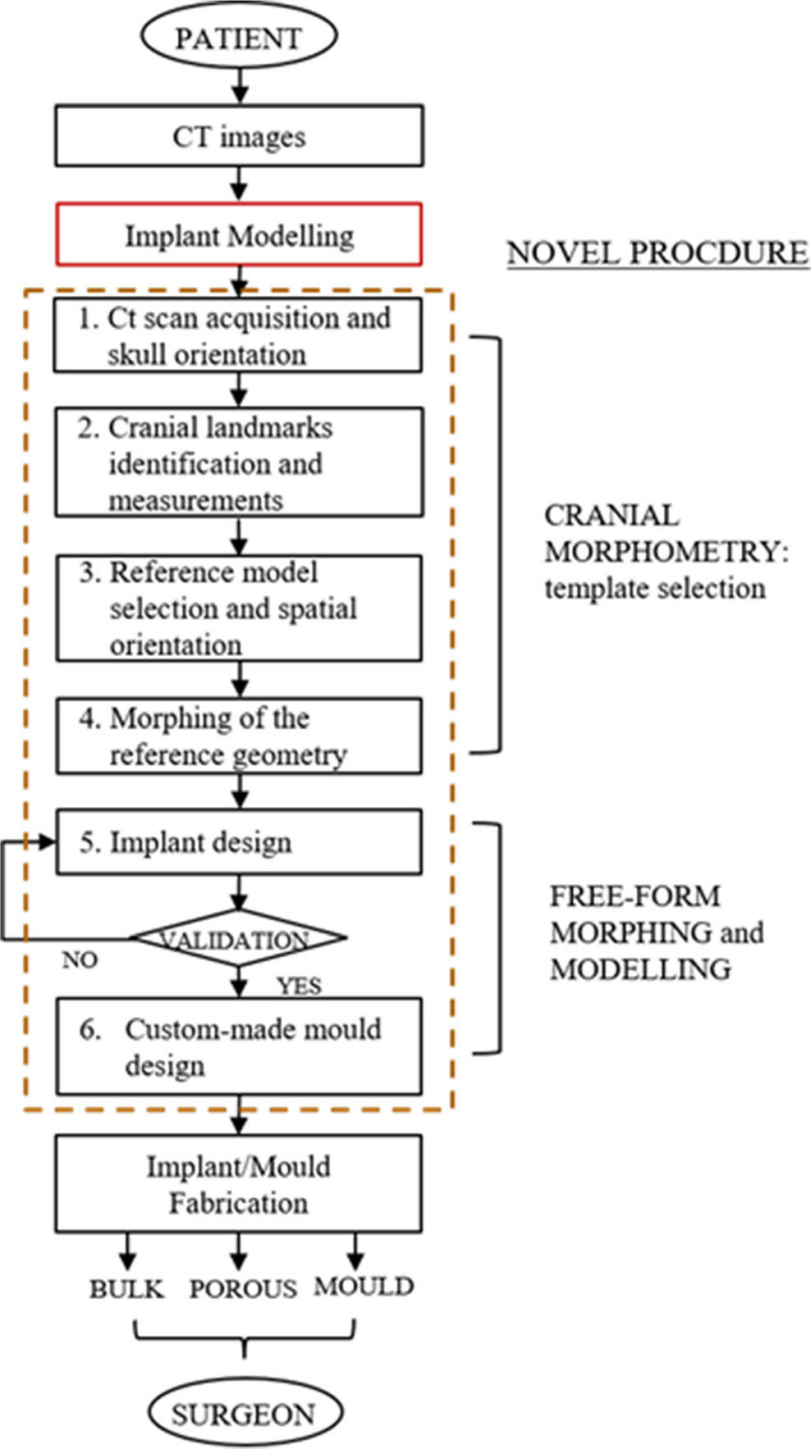
Workflow representing the steps of the novel design methodology

The proposed methodology for designing custom-made implants is shown in Fig. 1. The CT scan images of the patient’s skull are processed to retrieve the 3D anatomy useful for the implant modelling phase, which can be summarised in six steps. A dimensional validation procedure establishes the accuracy of the CAD model by evaluating its deviation from a physical prototype handcrafted by the surgeon. Once the deviation is lower than the acceptable tolerance, the 3D physical model of the implant can be fabricated (directly by using a 3D printing machine or indirectly, by using a custom-made mould) and sent to the surgeon.

Therefore, the principal points of the methodology are:

1. Acquisition of the CT images of the patient’s skull, reconstruction of the 3D anatomy and spatial orientation according to a reference coordinate system;
2. Computation of the main relevant measurements, according to cranial landmarks, useful for retrieving the best matching skull geometry;
3. Selection of the best fitting skull, from a CT skull database, to be used as a guide for the implant design and successive registration (alignment of the two skulls);
4. Morphing of the reference skull along the coronal and sagittal planes;
5. Implant design by using free-form tools to realise the anterior surface of the prosthesis, ensuring a smooth transition at the implant-bone interface;
6. Design of the custom-made mould for directly shaping the implant during surgery.

The following sections present the above-cited steps applied to a patient suffering from Apert syndrome.

### CT scan acquisition and skull orientation

Data acquisition for virtual modelling is performed through computed tomography imaging technique. The 2D slices are loaded into the medical imaging software *Mimics v. 12.11* by Materialise NV and segmented through a proper threshold. Then, the reconstructed 3D volumetric model of the skull is used for further modelling.

**Fig. 2:**
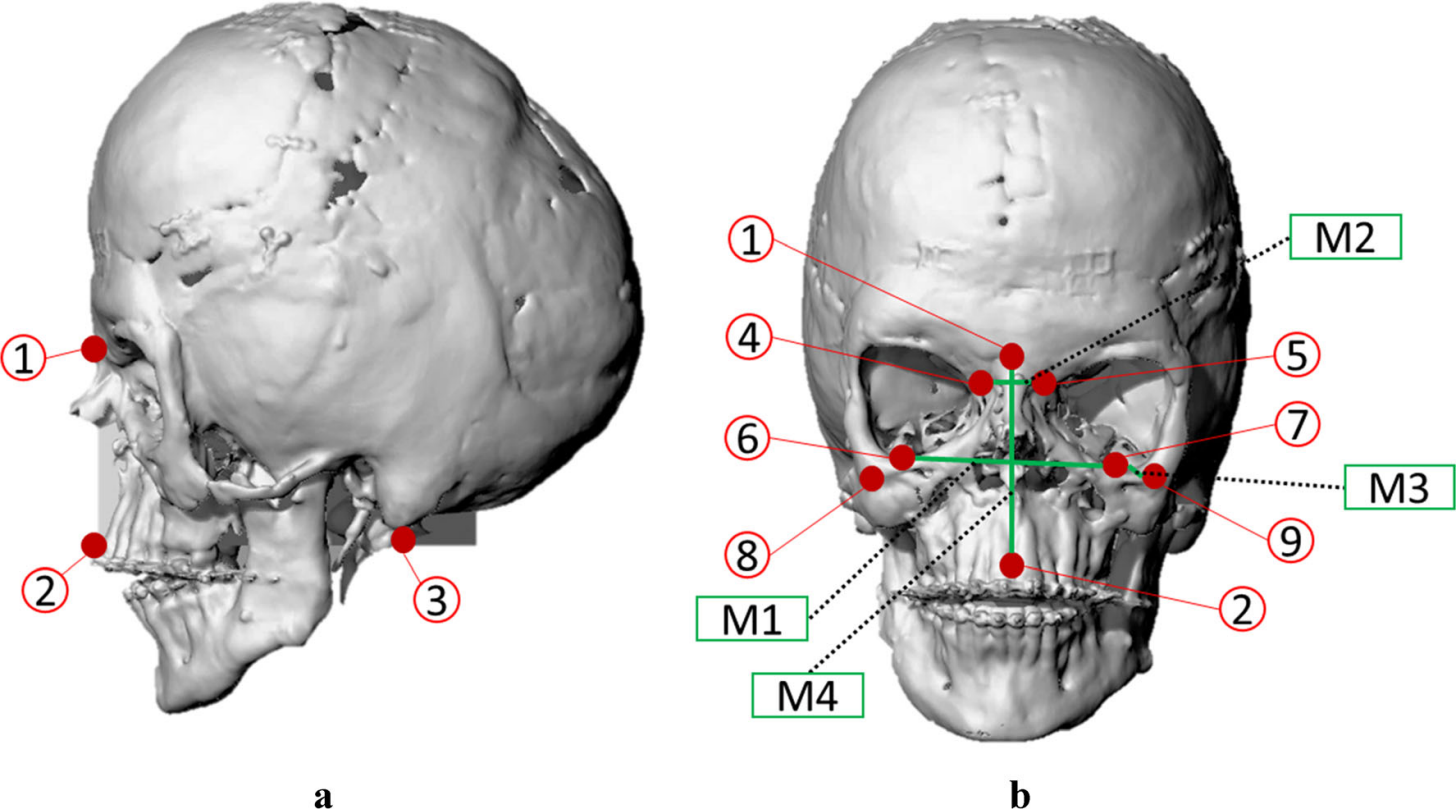
(**a**) Alignment of the sagittal plane of the skull to the Cartesian YZ plane. Point 1: Nasion, Point 2: Prosthion, Point 3: Opisthion. (**b**) Cranial landmarks (Points) and measurements (M) identified over the pathological skull. Point 1: Nasion, Point 2: Prosthion, Point 4: Maxillofrontal right, Point 5: Maxillofrontal left, Point 6: Orbital right, Point 7: Orbital left, Point 8: Porion right, Point 9: Porion left. M1: Interorbital distance (frontal plane, between points 6–7), M2: Maxillo-frontal distance (frontal plane, between points 4–5), M3: Porion-orbital distance (sagittal plane, between points 6–8, 7–9), M4: Nasion-prosthion distance (sagittal plane, between points 1–2)

The work of Farkas [12] on craniofacial measurements suggested the use of cranial landmarks to define a symmetry plane useful for the orientation and superimposition of two skull models. This plane vertically divides the skull into two corresponding sides. For the patient considered in this case, three bony unpaired craniometric landmarks were selected far enough to reduce the error in the alignment. The symmetry plane was generated by interpolation of the Nasion, Prosthion and Opisthion points (Fig. 2a) and overlapped to the sagittal YZ Cartesian plane.

### Cranial landmarks identification and measurements

The selection of the skull which best approximates the pathological one complies with an adequate algorithm whose inputs are the main relevant cranial measurements collected on several healthy skulls (over twenty in this work), indexed in a database according to demographic information such as age, sex and race. Although the literature based on anthropological investigations reports a series of cranial reference points to perform comparable measurements [13], the highly irregular surface of the patient’s skull, due to the pathology and the undergone surgical operations, made it difficult to identify those cranial points defined by the coronal and sagittal sutures. Thus, only the cranial points that most influence the geometry of the planned implant were selected. Figure 2b and Table 3 presents the cranial landmarks identified for the specific patient.

**Table 3:** Cranial landmarks definitions

LANDMARKS	DEFINITION
Maxillofrontal	The point of intersection of the anterior lacrimal crest and the frontomaxillary suture
Nasion	The midpoint of nasofrontal suture
Orbital	The lowest point in the margin of the orbit
Porion	The highest middle point on the margin of the external auditory meatus
Prosthion	The median point on the posterior margin of the occipital foramen

### Reference model selection and spatial orientation

The selection of the reference geometry from a database of skull samples was performed using a specific algorithm. First, geometries are filtered according to gender (i.e., *male* and *female*) and race (i.e., *Caucasoid*, *Mongoloid*, *Negroid*) of the selected patient. Age is not considered since forehead augmentation due by Apert syndrome is undergone in adult patients. Patient’s height is not considered since the reference skull will be morphed in the following step.

Each skull in the database gets a score that represents the measurement deviation from the pathological model. It is based on the Eq. (1), where *x* refers to each skull in the database:

1 $$ Score\_x=\frac{\sum_{i=1}^4\left|\frac{Mix- Mip}{Mip}\right|\ast pi}{4} $$

*i* refers to each measurement *M*. *M*_*1*_ is the interorbital distance, *M*_*2*_ is the inter maxillo-frontal distance, *M*_*3*_ is the distance between Porion and Orbital and *M*_*4*_ the distance between Nasion and Prosthion. *M*_*ip*_ stands for the measurements gained over the pathological skull (Fig. 2b). The score is a weighted mean where the weight is represented by *pi* and is calibrated according to the importance of the involved measurements. The frontal region is the most affected by the pathology. Thus, two-thirds of the weight is associated with those measurements (i.e., *p1 = p2 = 0.33*). The last two measurements have one-third of the weight hence *p3 = p4 = 0.17*. The lower the score, the higher the similarity.

### Morphing of the reference geometry

The cranial landmarks coordinates allow defining a spatial correspondence between the pathological skull and the template. An anatomical deformation process based on scale factors was performed to make the reference model suitable for the pathological skull.

A morphing procedure involved the deformation of the template skull on the frontal plane according to *M*_*1*_ and *M*_*2*_ because these measurements most influence the dimensions of the implants to be designed. Morphing on the sagittal plane was performed according to *M*_*3*_ and *M*_*4*_ to improve the matching between the two models. The scale factors to be used for the frontal and the sagittal morphing are calculated by averaging the ratio between the reference and pathological dimensions, *M*_*1*_ and *M*_*2*_ for the frontal morphing, *M*_*3*_ and *M*_*4*_ for the sagittal morphing (Table 4). In this manner, the orbital points overlap their correspondents on the patient skull, resulting in a more excellent matching in the region of interest (ROI).

**Table 4:** Scale factors to morph the reference skull

MEASUREMENTS	PATIENT mm	REFERENCE mm	SCALE FACTOR	AVG. SCALE FACTOR
M1. Interorbital distance (frontal plane)	72.4	61.9	1.17	1.37
M2. Maxillo-frontal distance (frontal plane)	27.4	17.3	1.58	
M3. Porion-orbital distance (sagittal plane)	66.7 (left) 60.1 (right)	73.0 (left) 75.6 (right)	0.90 (left), 0.80 (right), Average: 0.85	0.89
M4. Nasion-prosthion distance (sagittal plane)	64.8	69.4	0.93	

However, a third non-uniform scale could be necessary to extend the overlapping area up to the maxilla-frontal points. The control points of a cage fixed over the reference skull were edited to complete this deformation process. Then, free-form modelling was used to design the custom-made implant.

### Implant design

The personalisation and the accuracy of the implant depend on the following steps: curves drawing and extrusion, anterior surface realisation, morphing and sealing. First, the ROI is isolated by drawing two sets of curves over the template forehead to define its extension as suggested by the surgeon. The curves extrusion provides the contour of the ROI to be used as a guide for realising the anterior and posterior implant surfaces.

The ROI is split and isolated from the rest of the skull. All the penetrating layers of the same mesh are removed to obtain two layers representing the anterior and the posterior side of the implant. The latter corresponds to the forehead region of the patient; thus, only the anterior side of the implant must be realised.

**Fig. 3:**
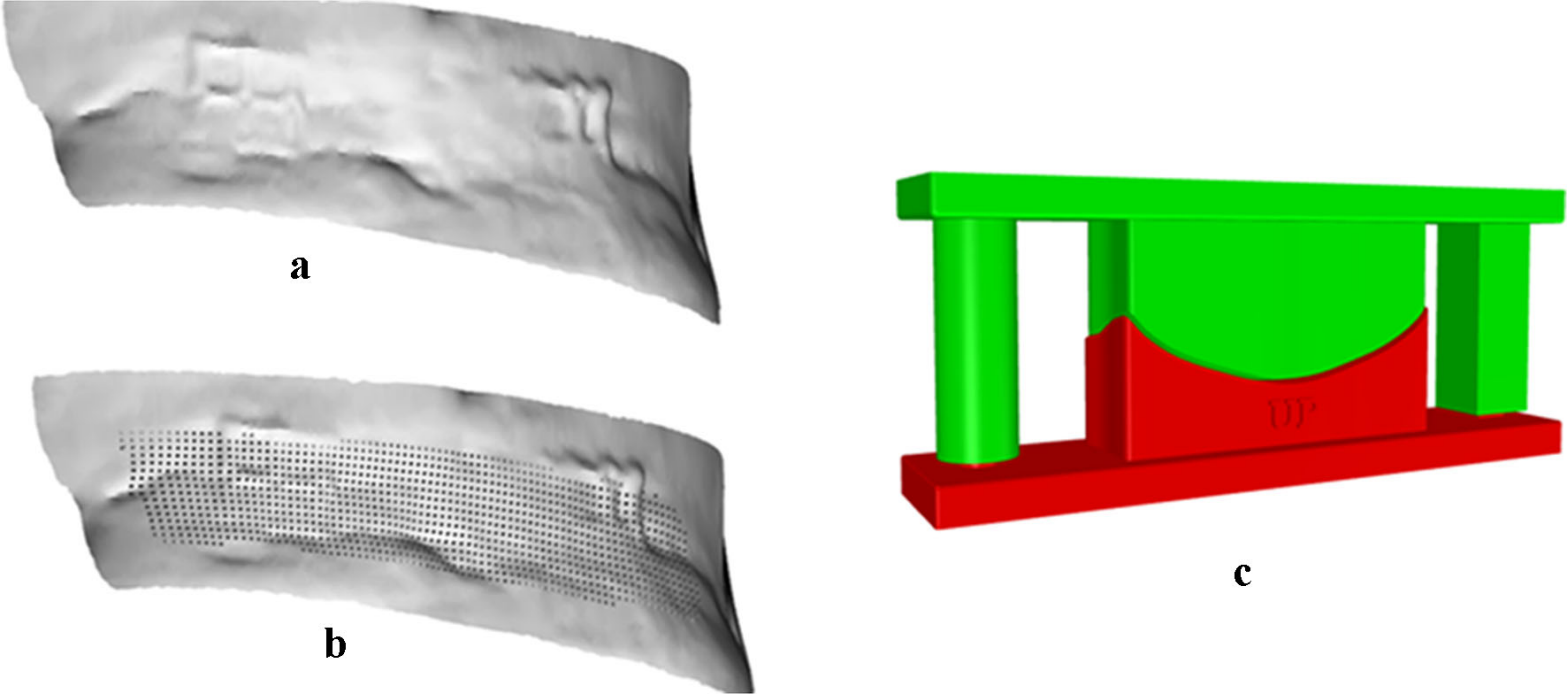
Custom-made implants designed for a patient suffering from Apert syndrome: full bulk (**a**) and porous (**b**) ones; Custom-made mould designed to shape a prefabricated implant (**c**)

To create the anterior surface of the implant, a radial array of 15 planes intersects both the ROI and the patient’s skull perpendicularly, providing a profile curve at each intersection. A line is tracked over each profile curve and edited by control points to be tangent to the borders of the implant, ensuring a continuous and smooth transition at the interface with the skull. The interpolation of all those cross-section curves will be used for creating the anterior surface of the implant, to be eventually adjusted in curvature, through control points, and sealed via Boolean union with the posterior surface, to get a watertight volume (Fig. 3a).

According to Syam et al. [14] the basic requirements for an implant material are the biocompatibility with tissues, without causing deleterious changes, and the ability to osseointegrate. Current trends in tissue engineering involve the design and manufacturing of biomimetic porous scaffolds based on the repetition of a unit cell, to control the mechanical properties and the permeability for nutrients transport [15]. Pores sized between 150 and 500 μm are just large enough to support the ingrowth of vascular tissues and cellular migration [16, 17]. According to Karageorgiou and Kaplan [17], cortical bone porosity should range between 3% and 12%. Porosity is considered to compensate for the biological inertia of Polyetheretherketone (PEEK), a biocompatible material widely used in spine surgery and cranioplasty [18]. Its mechanical properties are close to the human bone; it is lightweight, translucent and results in excellent aesthetic outcomes [19, 20].

Beside this bulk prosthesis, a porous version of the implant can be designed to resemble the actual bone tissue (Fig. 3b) and to promote osseointegration, thus preventing implant loosening or dislodgement [21]. It comprises a network of 500 μm square cross-section blinded-end channels [22], oriented along the sagittal plane and separated from each other by 500 μm, which cross the posterior side of the implant up to the central zone.

The geometrical accuracy of the implant will be estimated by a 3D comparison analysis with the scan of an implant physically shaped by the surgeon through plasticine. A negligible error entails the fabrication of the 3D models; otherwise, the implant design model should be improved.

### Custom-made mould design

To leave the surgeon free to choose the best implant material during surgery, a mould was designed (by using *Rhinoceros, by McNeel*) and realised. It is an equipment used by surgeons for realising the custom-made implant, during surgery, by deforming a sheet of a biocompatible material, selected according to patient’s needs, rather than using an implant already 3D printed.

The mould consists of a die and a punch (Fig. 3c) coupled in a pressure mechanism which allows the shaping of a prefabricated material. The active surface of the mould is designed starting from the previously defined bulk implant. The mechanism of insertion comprises two lateral guides, designed with a distinct shape to ensure correct alignment. The cylindrical guide has a radius of 5 mm, and the rectangular cross-section guide has a size of 10 mm × 10 mm. Friction in the coupling mechanism is avoided by considering an offset of 0.3 mm. Besides these mechanical requirements, some clinical aspects should be tested for realising an easy to handle and orient the mould to accommodate materials with a different thickness [6]. This kind of versatility is provided by a vertical gap of 2 mm between the guides and the pins. A 20 mm gap between the guides and the implant surface enables the surgeon to bend and contour the preformed material during surgery for a better adaptation to the defect site. The design of the mould can be improved by embedding a text over the die, providing the surgeon with a reference system useful for the implant orientation and positioning on the patient. The mould was thought to be used in the operating room. Thus sterilisation of the device must be ensured by a proper fabrication material (e.g. polyamide). The mould can be manufactured in polyamide PA 2200 (non-filled powder based on PA-12) using the SLS (Selective Laser Sintering) technique. Once realised, it is sterilised and intra-operatively used by the surgeon for shaping the custom-made implant, in combination with hot water.

## Results

### Custom-made implants design and manufacturing

**Fig. 4:**
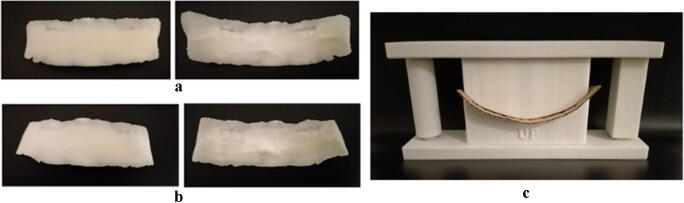
3D printed prototypes: (**a**) Full bulk volume front (left) and back (right); (**b**) Porous implant front (left) and back (right); (**c**) Custom-made mould

The design procedure presented in section 2 was used to design three custom-made implants for a patient suffering from Apert syndrome. The first designed implant was a full solid prosthesis (Fig. 4a) with dimensions 98 × 30 × 22 mm and a thickness ranging from 3 mm at the central region to 0.1 mm at the sides. The tapered edges ensure tangency at the interface with the skull to prevent any unpleasant feelings. Four Titan screws with a gauge of 1.5 mm and a length of 2–3 mm were thought for the implant fixation.

Porosity was introduced in the second version of the implant (Fig. 4b). However, the patient under study exhibited a forehead covered by a metallic mesh, due to previous surgical treatments, that compromised any chance of osteointegration. Therefore, the porous implant proposal was discarded by the surgeon. Besides, authors have thought initially to 3D print the PEEK implant, by using the Selective Laser Sintering (SLS) technology, which allows generating freeform and complex products. SLS printing has a dimensional tolerance of ±0.3% and a lower limit of ±0.3 mm. However, the cost of manufacturing this kind of implant is around 4.000–8.000€ depending on the size of the prosthesis. Most of this cost is related to the sterilisation required for a clean-room occupation. This cost is not affordable for most of the patients and even for the National Health System.

A solution was provided by Medpor Titan MAX sheet (by Stryker Craniomaxillofacial, Kalamazoo, MI), which has a reduced cost (824€) and a double-layer configuration providing the strength and radiopacity of titanium (for a post-operative imaging follow-up) and the flexibility of polyethylene to ensure easy modelling and shaping of the implant. The prefabricated Medpor Titan sheet was deformed intraoperatively according to the custom-made mould (Fig. 4c) based on the 3D CAD model of the full bulk implant. The overall mould dimensions were 190 mm × 40 mm × 80 mm, chosen to guarantee robustness and stability during the loading application.

### Custom-made implants validation

**Fig. 5:**
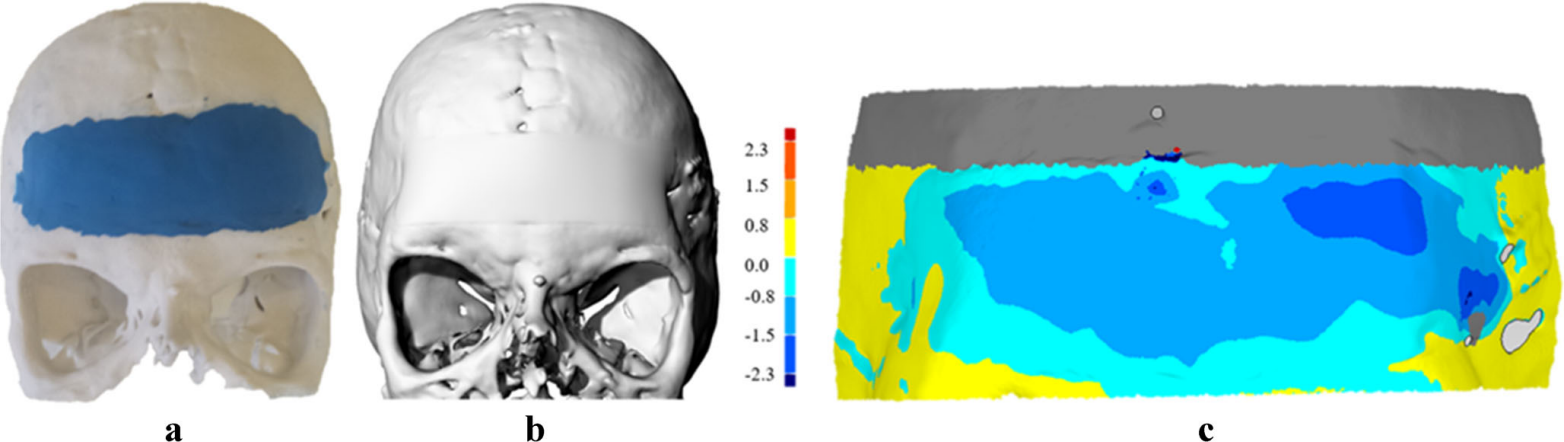
Outcomes evaluation: (**a**) A physical prototype, (**b**) CAD-based implant; c. 3D comparison analysis: the colour-coded map represents the deviation between the physical reference prototype and the test CAD implant

A validation procedure was carried out to assess the accuracy of the developed implant, by comparing the geometrical 3D CAD model of the implant (Fig. 5b) with the scan of a physical implant handcrafted by a maxillofacial surgeon through plasticine (Fig. 5a). The former was set as a test and the latter as a reference since it reproduces the ideal implant shape desired by the surgeon.

**Fig. 6:**
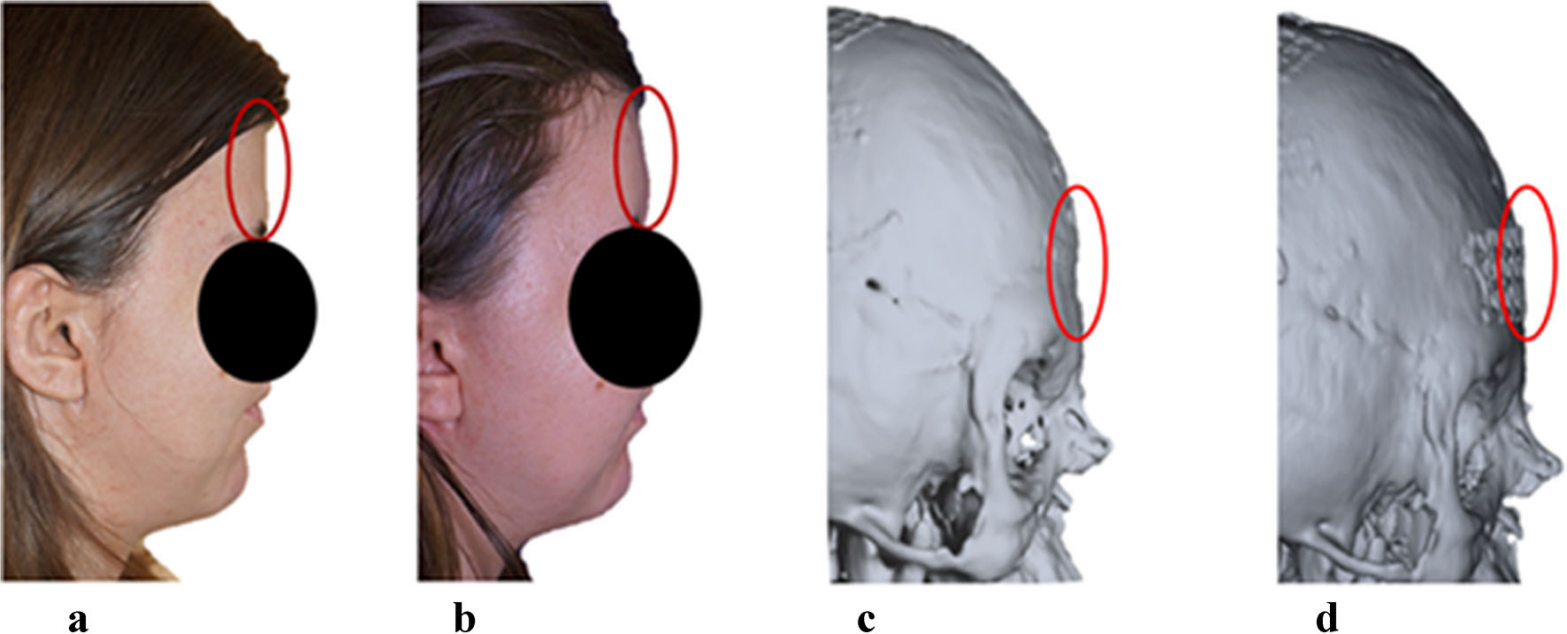
Pre (**a**) and post (**b**) surgical outcomes. The red circles highlight the affected region. Pre- (**c**) and post-(**d**) operative CT. Only the titanium mesh is visible because of the radiolucent nature of polyethylene

The outer surface of the implant was investigated to estimate its extension and deviation from the reference. The accuracy of the remodelled skull was tested in terms of average deviation in both positive and negative direction, maximum positive and maximum negative and mean of the absolute values. Figure 5c graphically represents the outcomes of the 3D analysis; the implant designed according to the proposed technique has an extension similar to the reference model. The average deviation is 0.2 mm in the inward direction and − 1.1 mm outwards. The maximum positive value is 0.4 mm, and the maximum negative value is −2.6 mm. The mean of the absolute values is 0.65 mm, thus meaning that the implant designed using the procedure has a thickness slightly smaller than the implant handcrafted by the surgeon, especially in the left side. Authors inferred that an average deviation of 0.65 mm could be considered as a negligible reconstruction error, consistent with the tolerance suggested by the surgeon, approximately 0.5 mm for maxillofacial surgeries [23]. The accuracy and the appearance of the designed cranial implant were confirmed by the post-treatment outcome shown in Fig. 6. Continuity and tangency at the interface with the bone are preserved. 3D-printing technologies allowed to get predictable outcomes resulting in minimal intraoperative implant contouring.

## Discussion

The design procedure, presented in section 2, was self-tested by the authors using the criteria listed in Table 1 and weights of Table 2. Hereunder the motivations that lead to the scores presented in Table 4.

- *Defect site: 9*. Apert syndrome determines bilateral and beyond the midface skull distortion;
- *Defect size: 6.* The designed implant has a surface larger than 25 cm^2^. Authors guess that the proposed methodology may also be used for implants more extensive than 200 cm^2^;
- *Degree of skull asymmetry: 9*. Apert syndrome is a congenital pathology that affects the whole skull since birth. Even if the patient considered in this paper had undergone several surgeries, his skull was still highly asymmetric;
- *Set-up required: 3*. The methodology was carried out after having prepared a database of reference geometries. CT images of twenty people (not affected by Apert) were considered for generating as many 3D geometries. Cranial landmarks and measurements were manually taken considering definitions provided in literature;
- *User intervention: 3.* The design procedure, even if systematic, is entirely manual, carried out by a technician using a commercial and general-purpose 3D CAD system (*Rhinoceros by McNeel*);
- *Operational time: 3*. The design procedure was performed without using any kind of 3D CAD modelling macros (e.g. skull orientation was manual);
- *Skills: 6.* The design procedure was performed by a junior biomedical engineer (one year after master’s degree) with medium expertise on CAD systems (lower than six months) and custom-made implants (never designed a custom-made implant before);
- *Quality: 9.* Through the design methodology proposed in this paper, it is possible to get an implant, whose borders are tangent and continue in curvature with the patient’s skull

According to these scores (Table 5), the authors’ methodology, in designing implants for people affected by Apert syndrome, got a weighted score of 6.90, which is higher than 6.45 (+7%) and 6.30 (+10%) respectively measured for *template-based* and free*-form modelling* methods.

**Table 5:** Evaluation of the proposed approach

Evaluation metrics	*Weight*	Proposed approach
Defect site	*0.00*	9 (0.00)
Defect size	*0.20*	6 (1.20)
Degree of skull asymmetry	*0.20*	9 (1.80)
Set-up required	*0.10*	3 (0.30)
User intervention	*0.05*	3 (0.15)
Operational time	*0.05*	3 (0.15)
Skills	*0.10*	6 (0.60)
Quality		
Curvature Tangency Continuity	*0.10* *0.10* *0.10*	9 (0.90) 9 (0.90) 9 (0.90
Total score	***1.00***	**66 (6.90)**

## Conclusions

The present paper outlined a methodological procedure to design a custom-made cranial implant for a patient affected by Apert syndrome. The developed procedure was intended for aesthetic reconstruction of a frontal bone deficiency on a highly deformed skull. The procedure comprises template deformation and anatomical reconstruction, based on the patient’s CT and a CT database of healthy skulls, to be used as reference geometry. Once selected the best fitting skull from CT database, by using anthropometric measurements, warping and deformation will be performed to adapt the region of interest to the target and to define the implant shape.

A dimensional validation of the implant design using the proposed procedure was carried out by a 3D comparison with a physical prototype, providing an acceptable result for maxillofacial surgeries. The design procedure was compared with other design methods used in surgery. Authors proved that the proposed method for the treatment of Apert syndrome, is about 7% and 10% respectively better than template-based and free-form modelling methods, the grounds of this work.

However, the manual identification of landmarks and the free-form modelling, two phases expected in this procedure, are time-consuming. In the future, the design procedure should be implemented in a 3D CAD system, by developing macros and plug-ins to make the processes of cranial landmarks identification, template retrieval and morphing, as automatic as possible and less user-dependent. By increasing the size of the CT skull database, it will be possible to improve the reliability of the design methodology (i.e., reduction of geometrical errors) and speed up the modelling phase (i.e., reduction of the morphing steps).
